# MRI-based multiregional radiomics for preoperative prediction of Ki-67 expression in meningiomas: a two-center study

**DOI:** 10.3389/fneur.2025.1554539

**Published:** 2025-07-24

**Authors:** Ming Luo, Guihan Lin, Duoning Chen, Weiyue Chen, Shuiwei Xia, Junguo Hui, Pengjun Chen, Minjiang Chen, Wangyang Ye, Jiansong Ji

**Affiliations:** ^1^Zhejiang Key Laboratory of Imaging and Interventional Medicine, Key Laboratory of Precision Medicine of Lishui City, Zhejiang Engineering Research Center of Interventional Medicine Engineering and Biotechnology, The Fifth Affiliated Hospital of Wenzhou Medical University, Lishui, China; ^2^Department of Neurosurgery, The Fifth Affiliated Hospital of Wenzhou Medical University, Lishui, China

**Keywords:** meningiomas, radiomics, intratumoral, peritumoral, Ki-67

## Abstract

**Background:**

High expression of Ki-67 in meningioma is significantly associated with higher histological grade and worse prognosis. The non-invasive and dynamic assessment of Ki-67 expression levels in meningiomas is of significant clinical importance and is urgently required. This study aimed to develop a predictive model for the Ki-67 index in meningioma based on preoperative magnetic resonance imaging (MRI).

**Methods:**

This study included 196 patients from one center (internal cohort) and 92 patients from another center (external validation cohort). Meningioma had to have been pathologically confirmed for inclusion. The Ki-67 index was classified as high (Ki-67 ≥ 5%) and low (Ki-67 < 5%). The internal cohort was randomly assigned to training and validation sets at a 7:3 ratio. Radiomics features were selected from contrast-enhanced T1-weighted MRI using the least-absolute shrinkage and selection operator and random forest methods. Then, we constructed a predictive model based on the identified semantic and radiomics features, aiming to distinguish high and low Ki-67 expression. The model’s performance was evaluated through internal cross-validation and validated in the external cohort.

**Results:**

Among the clinical features, peritumoral edema (*p* = 0.001) and heterogeneous enhancement (*p* = 0.001) were independent predictors of the Ki-67 index in meningiomas. The radiomics model using a combined 8 mm volume of interest demonstrated optimal performance in the training (area under the receiver operating characteristic curve [AUC] = 0.883) and validation (AUC = 0.811) sets. A nomogram integrating clinical and radiomic features was constructed, achieving an AUC of 0.904 and enhancing the model’s predictive accuracy for high Ki-67 expression.

**Conclusion:**

This study developed clinical-radiomic models to non-invasively predict Ki-67 expression in meningioma and provided a novel preoperative strategy for assessing tumor proliferation.

## 1 Introduction

Meningioma, rank as the second most common primary tumor of the central nervous system, accounting for approximately 39.7% of all intracranial tumors (1–3). Growth patterns differ among the different meningioma subtypes. Benign meningiomas have a five-year survival rate of 85.5% because of their slow growth. However, even after complete surgical resection, the five-year recurrence rate of benign meningiomas ranges from 7 to 25%, demonstrating a potential conversion to a high-grade subtype (2, 4). The resection status and histological grade significantly influence the management and prognosis of meningioma (5). The Ki-67 index is a critical biomarker of tumor proliferation in meningioma. Higher Ki-67 expression levels have been firmly established as a prognostic risk factor associated with unfavorable outcomes and increased risk of recurrence in affected individuals (6). Consequently, understanding the Ki-67 expression index in patients with meningioma is essential for managing risk stratification and clinical decision-making (7, 8).

In meningioma, the Ki-67 index is predominantly assessed in postoperative specimens using the immunohistochemical (IHC) technique. However, it’s invasive nature and reliance on retrospective tissue sampling restrict its clinical utility for preoperative therapeutic planning and longitudinal progression monitoring. Many patients with meningioma require long-term follow-up, and since Ki-67 expression is a valuable prognostic marker, a tailored follow-up strategy is necessary to balance patient well-being with effective disease management, as traditional imaging methods struggle to assess tumor proliferation accurately (9). As a result, there is still an urgent clinical need for a readily accessible method for assessing the Ki-67 index. Previous studies indicate that preoperative magnetic resonance imaging (MRI) features, such as tumor heterogeneity on enhanced T1 images, apparent diffusion coefficient images, irregular tumor shape, and peritumoral brain edema, are valuable for evaluating the grade and histopathological characteristics of meningiomas (10–13). Radiomics incorporates automated calculation methods into precise quantitative analysis techniques, and applies them to imaging diagnosis, establishing classification models through analysis and screening (14). It streamlines the diagnostic process, minimizes the necessity for invasive procedures, and accelerates treatment planning. As radiomics technology evolves, its application in predicting the pathological grade and clinical prognosis of brain tumors has gained increasing recognition (12, 15). A focused systematic review and meta-analysis evaluated the performance of MRI-derived radiomics models for predicting Ki-67 status, demonstrating the growing interest and promising results in this area. These studies provide a foundation for our research, which leverages a multiregional radiomics approach based on MRI to preoperatively predict Ki-67 expression in meningiomas (16).

This study aimed to derive radiomics features from the peritumoral and intratumoral regions, providing valuable insights into predicting the Ki-67 expression status of patients with meningioma. To achieve this aim, we developed and validated a model that integrated clinical semantic features with radiomics features using machine learning algorithms, intending to non-invasively predict the Ki-67 expression status of these patients.

## 2 Materials and methods

### 2.1 Patients

**Figure 1:**
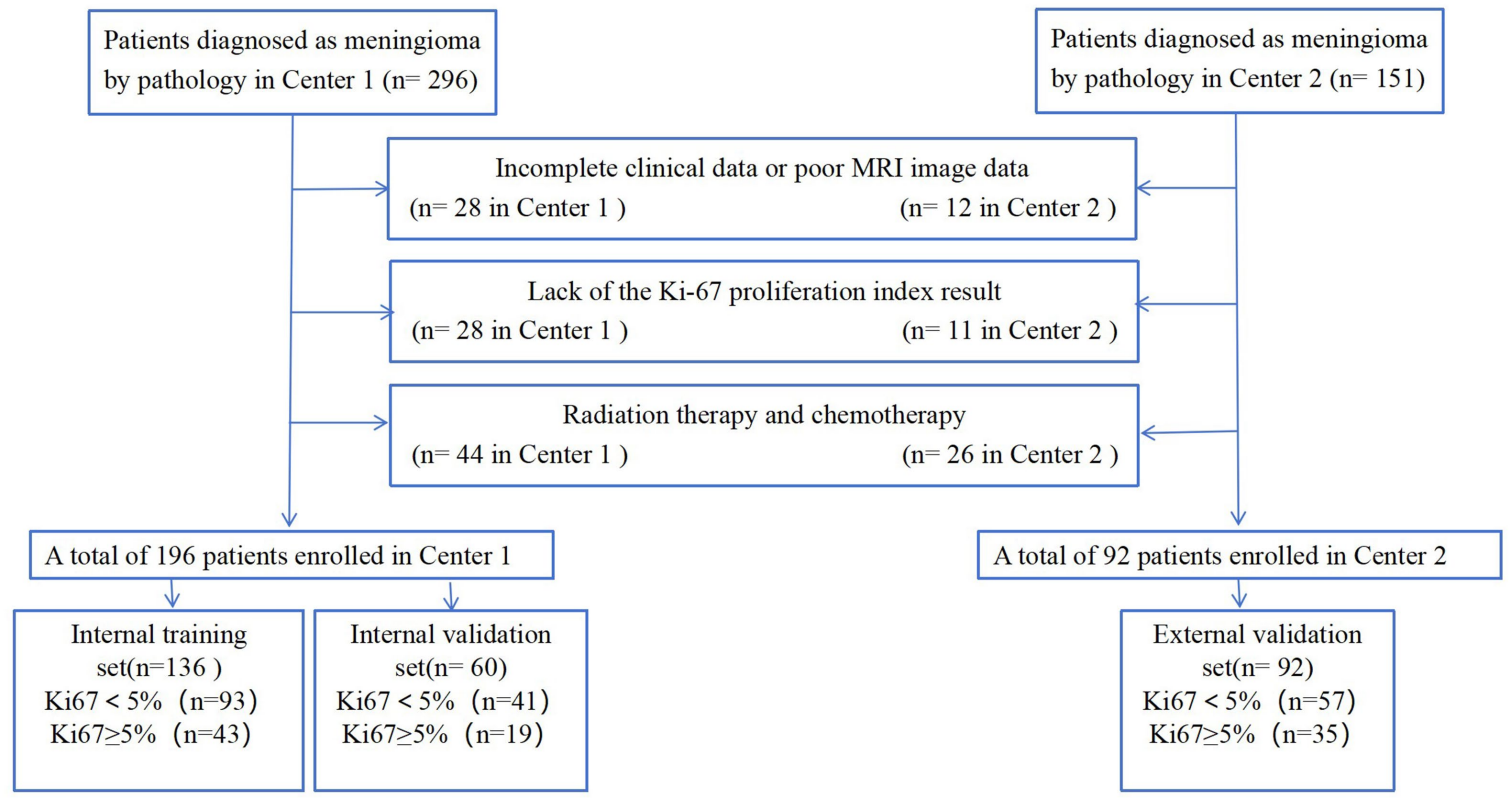
Flowchart of the patient selection criteria.

This study initially enrolled 296 patients from the Fifth Affiliated Hospital of Wenzhou Medical University (Center 1) and 151 from the Sixth Affiliated Hospital of Wenzhou Medical University (Center 2) between November 2009 and May 2023. The meningioma diagnoses were confirmed through surgical pathology. The inclusion criteria were as follows: (1) patients who underwent MRI plain and enhanced scans 1 month before surgery, with complete clinical data; (2) patients who had not received radiotherapy or any other treatment before the MRI scan and had no history of head surgery; (3) patients with confirmed postoperative pathology and a determined Ki-67 index. The exclusion criteria were as follows: (1) patients with incomplete clinical data or poor MRI image quality; (2) patients lacking Ki-67 proliferation index results; (3) patients who had undergone radiation therapy and chemotherapy. Ultimately, 288 patients (196 from Center 1 and 92 from Center 2) were included in this study. The data from Center 1 were used for model development, while the data from Center 2 were used for external validation. Lesions from Center 1 were randomly assigned into training (*n* = 136) and validation (*n* = 60) sets at a ratio of 7:3. The detailed patient enrollment process is outlined in Figure 1. All study protocols and procedures were conducted in compliance with the Declaration of Helsinki. The requirement for informed consent from patients was waived due to the retrospective nature of this study.

### 2.2 MRI protocol

Preoperative MRI examinations were performed at two hospitals. At Center 1, MRI examinations were performed on a 3 T scanner (Magnetom Skyra; Siemens Healthineers, Erlangen, Germany). The imaging protocols included the following sequences: T1-weighted imaging (T1WI; repetition time [TR]/echo time [TE] = 2,540/9.4 ms, matrix size = 384 × 269), T2-weighted imaging (T2WI; TR/TE = 3,700/100 ms, matrix size = 448 × 311), fluid-attenuated inversion recovery (FLAIR; TR/TE = 7,000/86 ms, matrix size = 384 × 230), and contrast-enhanced T1WI (CE-T1WI; TR/TE = 149/3.4 ms, matrix size = 480 × 381). All images were taken with a field of view (FOV) of 230 × 230 mm, a slice thickness of 4 mm, and no interslice gaps. Contrast-enhanced MRI scans were performed following a bolus dose of 0.2 mL/kg of a contrast agent.

At Center 2, MRI examinations were performed using a 3 T scanner (Discovery 750; GE Healthcare, Chicago, IL, United States). The detailed protocol included the following sequences: T1WI (TR/TE = 2,200/11 ms, matrix = 320 × 256), T2WI (TR/TE = 3,900/87 ms, matrix = 512 × 384), FLAIR (TR/TE = 6,800/113 ms, matrix = 384 × 256), and CE-T1WI (TR/TE = 294/3.6 ms, matrix = 384 × 256). All images were taken with a FOV of 230–240 × 230–240 mm, a slice thickness of 5 mm, and a gap of 0.5 mm. The CE-T1WI images were acquired after a bolus dose of 0.2 mL/kg of a contrast agent.

### 2.3 Data collection

The patients’ baseline clinical data were collected, including sex, age, headache history, history of epilepsy, history of malignancy, diabetes mellitus, hypertension, history of allergies, history of alcohol abuse, and history of tobacco addiction, along with their laboratory test results, including leucocyte, neutrophil, lymphocyte, and monocyte counts; plasma fibrinogen level; and serum albumin level. Two attending physicians with 5 and 11 years of neuroimaging diagnostic experience were assigned to analyze the images using a post-processing workstation. Their evaluations primarily focused on tumor location, morphology, peritumoral edema, necrosis, and enhancement characteristics. In cases where they disagreed on the interpretation results, consensus was achieved through collaborative consultation.

### 2.4 Pathological analysis

The surgical tissue samples were initially fixed in a 10% formaldehyde solution, then dehydrated and embedded in paraffin for IHC staining of Ki-67. Under microscopic examination, cells exhibiting dark brown granules within their nuclei were identified as Ki-67+. The Ki-67 index was determined by calculating the percentage of Ki-67 + cells relative to the total cell count. A Ki-67 index of <5% was classified as low expression, and a Ki-67 index of ≥5% was classified as high expression (11, 12, 17).

### 2.5 Image segmentation and feature extraction

Delineation of the tumor volume of interest (VOI) and segmentation for radiomics feature extraction were conducted using the Radcloud platform. Before extracting features, all images were resampled to a uniform voxel size of 1 × 1 × 1 mm^3^ using B-Spline interpolation, ensuring consistent slice thickness and preserving rotational symmetry. Additionally, to address differences in pixel brightness between two distinct MRI machines, the intensity of gray levels in all image datasets was normalized to a range of 0–255 after removing pixels with anomalous values. The normalization pipeline involves applying N4 bias field correction to address intensity inhomogeneities followed by Z-score normalization to standardize the data scale and mitigate feature variations caused by intensity differences. CE-T1WI images were used for delineation, carefully excluding non-brain tissues such as the skull. Image normalization was conducted before feature selection to minimize grayscale variability and individual differences. Initially, an attending physician with 5 years of neuroimaging diagnostic experience manually traced the tumor boundaries layer-by-layer to define the intratumoral VOI. The peritumoral VOI was generated using automated software, expanding at intervals of 2, 4, 6, 8, and 10 mm from the tumor outline. After delineation, another attending physician with 11 years of neuroimaging diagnostic experience reviewed the VOIs. In cases of inconsistency, the two physicians reached a consensus through discussion. Both were blinded to patient groupings. The extracted radiomic features included first-order statistics, morphological features, and texture features. First-order statistics quantitatively describe the intensity distribution of voxels within MRI images. Morphological features represent three-dimensional aspects, reflecting the shape and size of the lesion. Texture features assess heterogeneity within the VOI.

### 2.6 Feature filtering and machine learning classifier building

**Figure 2:**
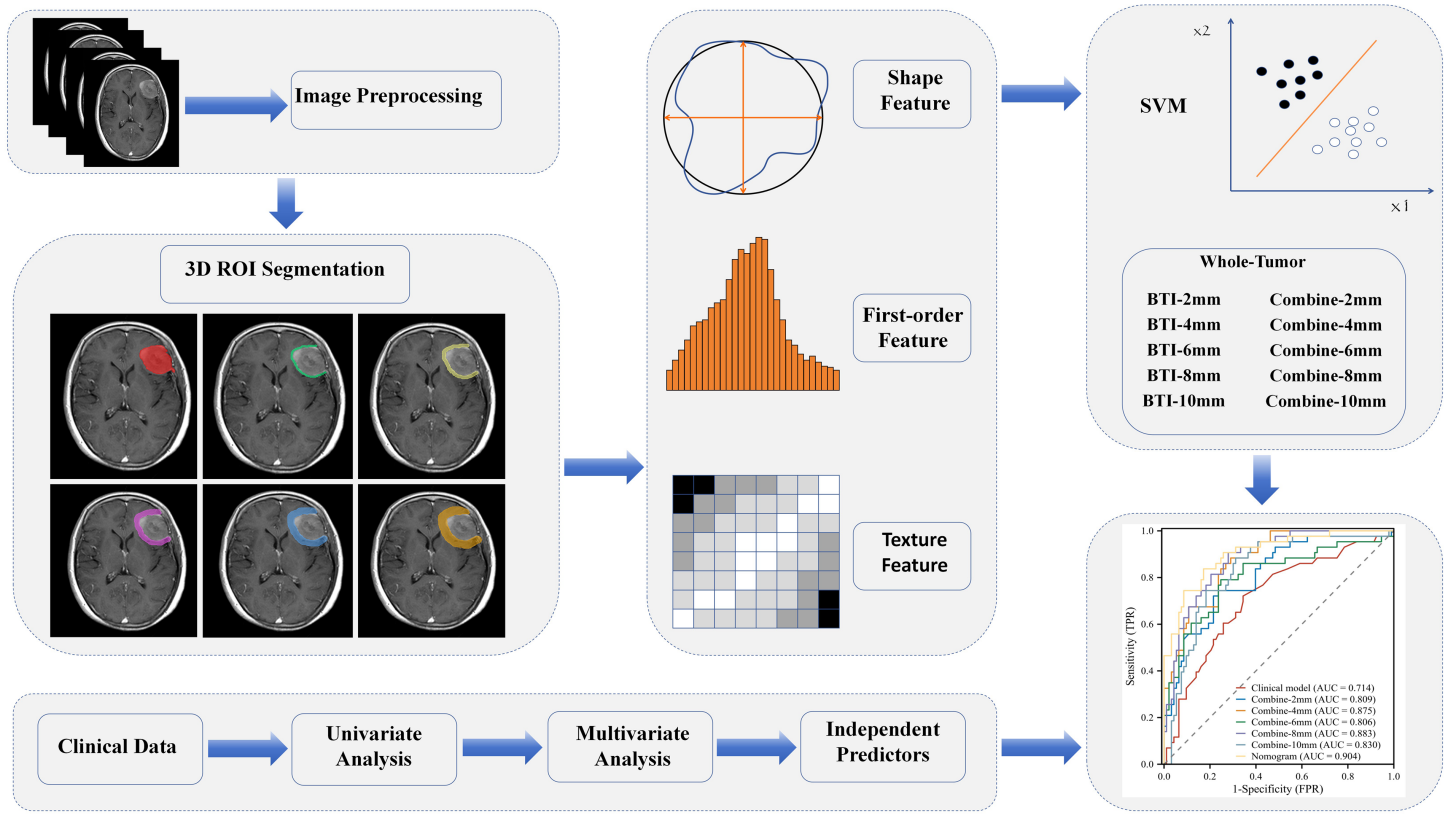
Study workflow.

Radiomics features were screened using variance threshold, SelectKBest method, and least absolute shrinkage and selection operator (LASSO) logistic regression. A threshold of 0.8 was set, and features with variance below this value were excluded. Using the SelectKBest method, features with a *p*-value < 0.05 were retained. The LASSO algorithm was used for cross-verification, identifying the optimal radiomics features characterized by non-zero regression coefficients. A series of distinct models were constructed using a Random Forest (RF) classifier: (1) a clinical semantic model; (2) a tumoral radiomics model; (3) five peritumoral radiomics models at incremental distances of 2, 4, 6, 8, and 10 mm from the tumor outline; (4) five combined radiomics models integrating intratumoral features with five widths of peritumoral features; and (5) a comprehensive model that merged clinical semantic and radiomics features. Clinical features with a *p*-value <0.05 in the univariate analyses were included in the multivariate logistic regression to screen for clinical risk factors and establish clinical models. Ultimately, a comprehensive model integrating clinical risk factors and the best radiomic features was constructed, and a nomogram was developed. An overview of the clinical and radiomic feature analyses is provided in Figure 2.

The Rad-score signature was generated using a Random Forest (RF) classifier to combine the radiomics features into a single signature. The RF classifier was trained using the filtered radiomics features and the corresponding patient outcomes (high or low Ki-67 expression). The resulting Rad-score signature was then used as an input feature in the nomogram model.

### 2.7 Statistical analysis

All statistical analyses were conducted using Python (version 3.7.6) and the R (version 4.3.1) statistical software. The Kolmogorov–Smirnov test was used to assess the normality of continuous variables. Student’s t-test was used to compare normally distributed continuous variables, the Mann–Whitney *U* test was used to compare non-normally distributed continuous variables, and the chi-square test was used to compare categorical variables. The R packages used in this study included “glmnet” (for LASSO regression), “rms” (for logistic regression analysis and calibration curves), “rmda” (for decision curve analysis [DCA]), and “PredictABEL” (for calculating the net reclassification improvement [NRI] and integrated discrimination improvement [IDI]). Receiver operating characteristic (ROC) analysis was conducted using MedCalc, and the DeLong test was used to compare the differences in the area under the ROC curve (AUC) between models. All tests were two-tailed, and a *p* < 0.05 was considered statistically significant.

## 3 Results

### 3.1 Patient characteristics and clinical model construction

The clinical and radiological characteristics of the enrolled patients are summarized in Table 1. The training set comprised 136 patients and the validation set comprised 60 patients. In both the training and validation sets, the sex distribution, histories of chronic conditions (e.g., malignancy, diabetes, and hypertension), and laboratory research indices did not vary substantially between the high and low Ki-67 expression groups. However, in both cohorts, patients with peritumoral edema and those with heterogeneous enhancement were significantly more common in the high Ki-67 expression group (all *p* < 0.05).

**Table 1:** The clinical and radiological characteristics of the enrolled patients.

Characteristic	Internal training set		*p* value	Internal validation set		*p* value	External validation set		*p* value
	Ki-67 < 5% (*n* = 93)	Ki-67 ≥ 5% (*n* = 43)		Ki-67 < 5% (*n* = 41)	Ki-67 ≥ 5% (*n* = 19)		Ki-67 < 5% (*n* = 57)	Ki-67 ≥ 5% (*n* = 35)	
Age (year, mean ± SD)	55.30 ± 9.24	60.14 ± 10.64	0.008	52.44 ± 8.69	58.79 ± 13.44	0.032	59.18 ± 9.10	57.77 ± 8.75	0.468
Gender			0.130			0.839			0.170
Male	21 (22.6)	15 (34.9)		14 (34.1)	7 (36.8)		15 (26.3)	14 (40.0)	
Female	72 (77.4)	28 (65.1)		27 (65.9)	12 (63.2)		42 (73.7)	21 (60.0)	
History of Headache			0.566			0.872			0.597
Negative	58 (62.4)	29 (67.4)		25 (61.0)	12 (63.2)		42 (73.7)	24 (68.6)	
Positive	35 (37.6)	14 (32.6)		16 (39.0)	7 (36.8)		15 (26.3)	11 (31.4)	
History of epilepsy			0.243			0.663			0.864
Negative	83 (89.2)	41 (95.3)		35 (85.4)	17 (89.5)		55 (96.5)	34 (97.1)	
Positive	10 (10.8)	2 (4.7)		6 (14.6)	2 (10.5)		2 (3.5)	1 (2.9)	
History of malignancy			0.530			0.492			0.615
Negative	86 (92.5)	41 (95.3)		40 (97.6)	19 (100.0)		55 (96.5)	33 (94.3)	
Positive	7 (7.5)	2 (4.7)		1 (2.4)	0 (0.0)		2 (3.5)	2 (5.7)	
History of diabetes			0.511			0.558			0.255
Negative	89 (95.7)	40 (93.0)		37 (90.2)	18 (94.7)		53 (93.0)	30 (85.7)	
Positive	4 (4.3)	3 (7.0)		4 (9.8)	1 (5.3)		4 (7.0)	5 (14.3)	
History of hypertension			0.263			0.303			0.710
Negative	65 (69.9)	34 (79.1)		27 (65.9)	15 (78.9)		38 (66.7)	22 (62.9)	
Positive	28 (30.1)	9 (20.9)		14 (34.1)	4 (21.1)		19 (33.3)	13 (37.1)	
History of allergic			0.164			0.139			0.864
Negative	91 (97.8)	40 (93.0)		41 (100.0)	18 (94.7)		55 (96.5)	34 (97.1)	
Positive	2 (2.2)	3 (7.0)		0 (0.0)	1 (5.3)		2 (3.5)	1 (2.9)	
Alcohol abuse			0.859			0.079			0.466
Negative	88 (94.6)	41 (95.3)		35 (85.4)	19 (100.0)		53 (93.0)	31 (88.6)	
Positive	5 (5.4)	2 (4.7)		6 (14.6)	0 (0.0)		4 (7.0)	4 (11.4)	
Cigarette addict			0.383			0.150			0.716
Negative	81 (87.1)	35 (81.4)		33 (80.5)	18 (94.7)		32 (56.1)	21 (60.0)	
Positive	12 (12.9)	8 (18.6)		8 (19.5)	1 (5.3)		25 (43.9)	14 (40.0)	
Tumor morphology			0.030			0.432			0.636
Regular	71 (76.3)	25 (58.1)		28 (68.3)	11 (57.9)		37 (64.9)	21 (60.0)	
Irregular	22 (23.7)	18 (41.9)		13 (31.7)	8 (42.1)		20 (35.1)	14 (40.0)	
Peritumoral edema			0.001			0.017			0.002
Negative	62 (66.7)	19 (44.2)		32 (78.0)	9 (47.4)		45 (78.9)	16 (45.7)	
Positive	31 (33.3)	24 (55.8)		9 (22.0)	10 (52.6)		12 (21.1)	19 (54.3)	
Necrosis			0.012			0.498			0.299
Negative	87 (93.5)	34 (79.1)		37 (90.2)	16 (84.2)		34 (59.6)	17 (48.6)	
Positive	6 (6.5)	9 (20.9)		4 (9.8)	3 (15.8)		23 (40.4)	18 (51.4)	
Enhancement			0.001			<0.001			0.001
Uniform	72 (77.4)	21 (48.8)		32 (78.0)	5 (26.3)		48 (84.2)	18 (51.4)	
Heterogeneous	21 (22.6)	22 (51.2)		9 (22.0)	14 (73.7)		9 (15.8)	17 (48.6)	
Tumor location			0.504			0.784			0.546
Convexity	55 (59.1)	29 (67.4)		28 (68.3)	13 (68.4)		37 (64.9)	26 (74.3)	
Skull base	37 (39.8)	13 (30.2)		12 (29.3)	6 (31.6)		9 (15.8)	8 (22.9)	
Ventricle	1 (1.1)	1 (2.3)		1 (2.4)	0 (0.0)		1 (1.8)	1 (2.9)	
Leucocyte count	6.49 ± 2.62	6.42 ± 3.37	0.891	7.39 ± 3.46	7.78 ± 4.15	0.709	8.172.67	7.92 ± 4.21	0.468
Neutrophil count	4.16 ± 2.60	4.25 ± 3.35	0.855	5.15 ± 3.40	5.58 ± 3.92	0.669	4.85 ± 2.41	4.52 ± 4.34	0.641
Lymphocyte count	1.76 ± 0.58	1.61 ± 0.53	0.168	1.59 ± 0.48	1.60 ± 0.55	0.976	1.61 ± 0.49	1.56 ± 0.48	0.581
Monocyte count	0.40 ± 0.17	0.37 ± 0.15	0.356	0.50 ± 0.27	0.46 ± 0.23	0.537	0.44 ± 0.22	0.43 ± 0.14	0.733
Plasma fibrinogen	2.91 ± 0.83	3.07 ± 0.84	0.320	3.47 ± 0.93	3.39 ± 0.75	0.748	3.28 ± 1.01	3.16 ± 0.91	0.544
Serum-albumin	40.66 ± 3.94	38.92 ± 3.48	0.014	40.44 ± 3.45	39.70 ± 3.93	0.462	35.98 ± 9.10	57.77 ± 8.75	0.067

*P* represents the difference in each clinicopathological variable between the high and low Ki-67 expression groups. The chi-square test was used to compare the difference in categorical variables (all variables except age), while a Student’s t-test was used to compare the difference in age.

16 clinical features and 5 radiological features were utilized to construct the clinical model. Among the clinical parameters, peritumoral edema (odds ratio [OR] = 3.733, 95% confidence interval [CI] = 1.744–7.991, *p* = 0.001) and heterogeneous enhancement (OR = 3.592, 95% CI = 11.662–7.762, *p* = 0.001) were identified as significant predictors of high Ki-67 expression (Table 2). These results suggested that patients presenting with these radiological features were more likely to have a higher Ki-67 index.

**Table 2:** Univariate and multivariate analyses for predicting Ki-67 expression.

Features	Univariate logistic	*p*	Multivariate logistic	*p*
	OR (95% CI)		OR (95% CI)	
Age	1.052 (1.012–1.094)	0.010		
Gender	1.837 (0.831–4.061)	0.133		
History of Headache	0.800 (0.373–1.717)	0.567		
History of epilepsy	0.405 (0.085–1.934)	0.257		
History of malignancy	0.599 (0.119–3.013)	0.534		
History of diabetes	1.669 (0.357–7.806)	0.515		
History of hypertension	0.615 (0.261–1.449)	0.266		
History of allergic	3.412 (0.549–21.219)	0.188		
Alcohol abuse	0.858 (0.160–4.612)	0.859		
Cigarette addict	1.543 (0.580–4.105)	0.385		
Tumor morphology	2.324 (1.074–5.028)	0.032		
Peritumoral edema	3.733 (1.744–7.991)	0.001	2.943 (1.329–6.516)	0.008
Necrosis	3.838 (1.269–11.605)	0.017		
Enhancement	3.592 (11.662–7.762)	0.001	2.722 (1.211–6.120)	0.015
Tumor location	0.765 (0.374–1.562)	0.462		
Leucocyte count	0.991 (0.871–1.127)	0.890		
Neutrophil count	1.012 (0.893–1.147)	0.854		
Lymphocyte count	0.626 (0.321–1.220)	0.169		
Monocyte count	0.312 (0.026–3.714)	0.357		
Plasma fibrinogen	1.243 (0.810–1.907)	0.320		
Serum-albumin	0.885 (0.800–0.980)	0.018		

OR, odds ratio; CI, Confidence interval.

### 3.2 Model construction

**Figure 3:**
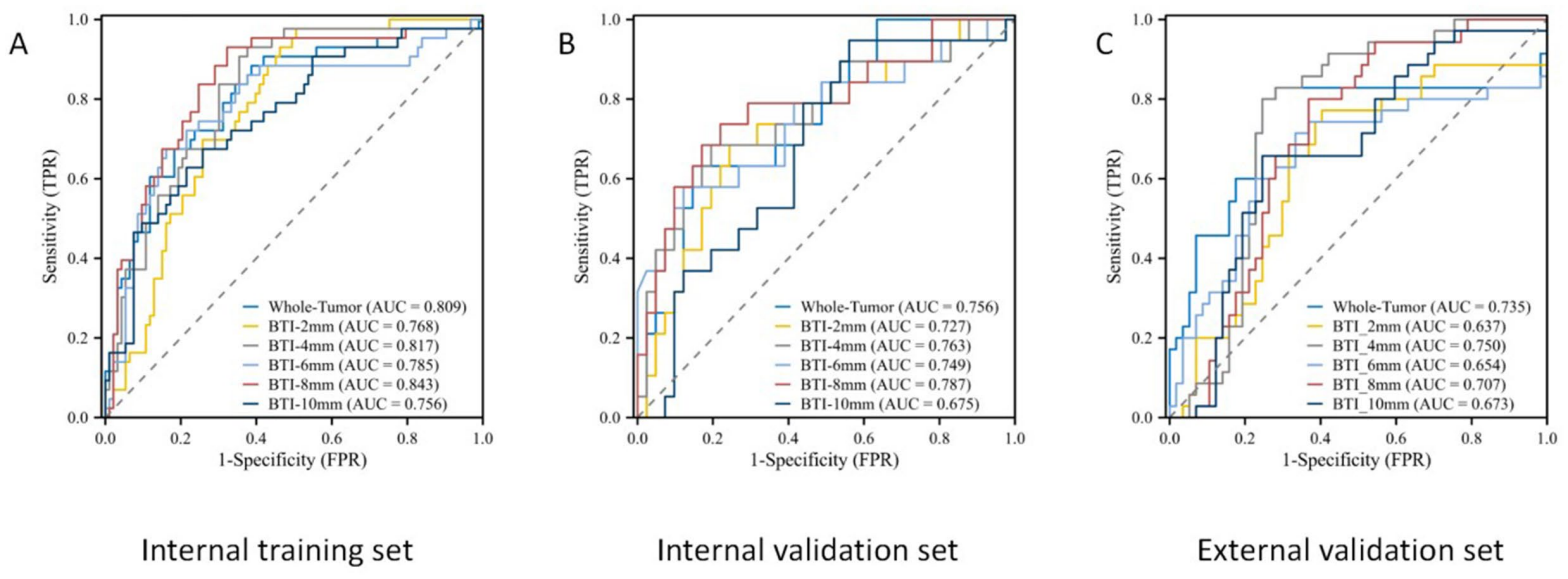
The ROC curves for the tumoral and peritumoral radiomics model. ROC curves in the **(A)** internal training set, **(B)** internal validation set, and **(C)** external validation set.

**Figure 4:**
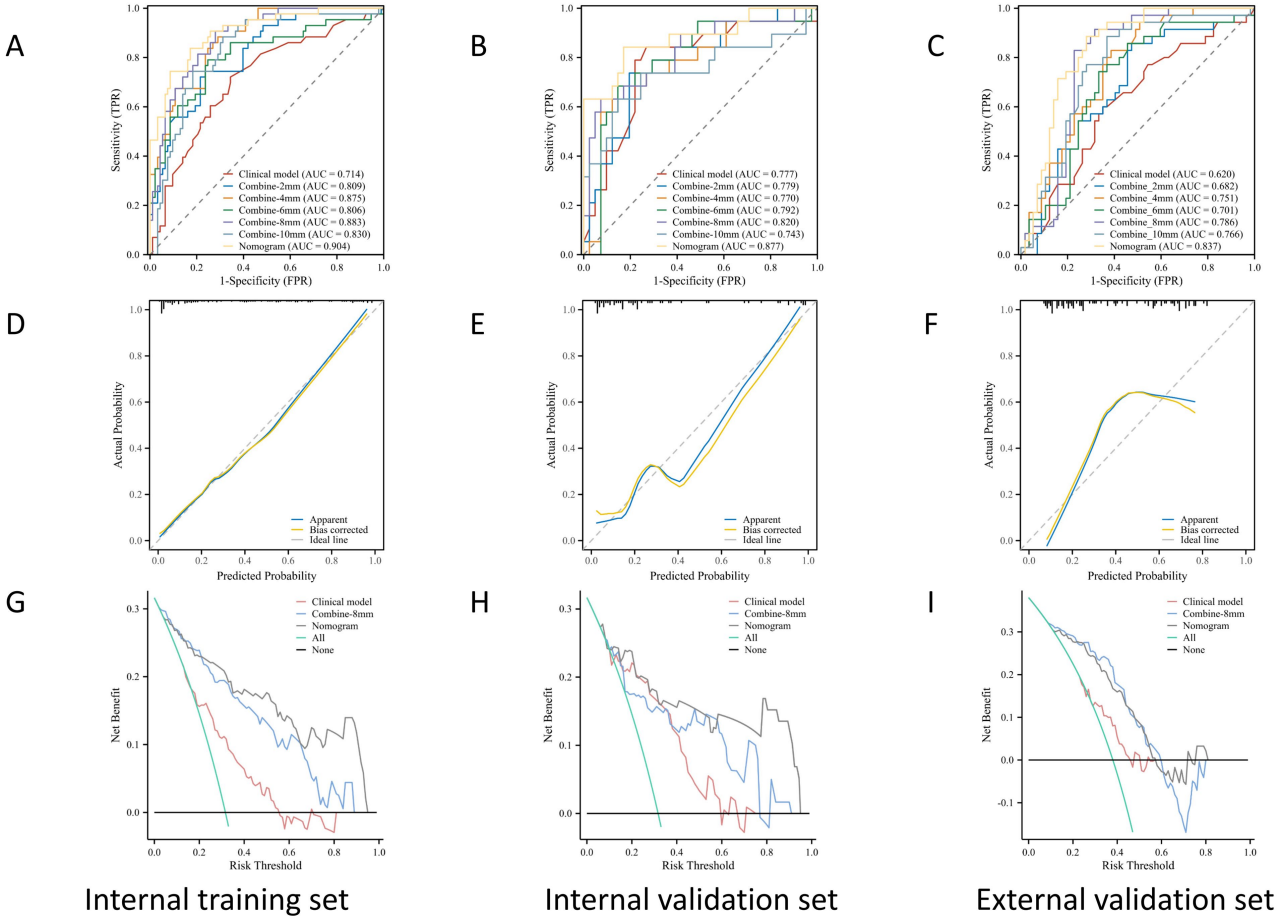
The ROC, calibration, and DCA curves. ROC curves in the **(A)** internal training set, **(B)** internal validation set, and **(C)** the external validation set. Calibration curves in the **(D)** internal training set, **(E)** internal validation set, and **(F)** external validation set. The prediction results were consistent with the diagonal line, indicating the accurate prediction results. The DCA curves for the diagnostic nomogram in the **(G)** internal training set, **(H)** internal validation set, and **(I)** external validation set.

The ROC curves for the tumoral and peritumoral radiomics models are shown in Figure 3, illustrating the diagnostic performance of the model in distinguishing patients with high and low Ki-67 expression. Multivariate logistic analysis showed that peritumoral edema, heterogeneous enhancement, and 8 mm-based radiomics signature were independent predictors of high Ki-67 expression. These predictors were incorporated into a comprehensive model that merged clinical semantic and radiomics features. The radiomics signature was derived from the combined-8 mm (the whole tumor plus 8 mm peritumoral area) model, which demonstrated superior performance compared to other peritumoral widths (2, 4, 6, 10 mm). Then, these independent predictors were incorporated into a model and presented as a nomogram. The calibration curves and DCA for the diagnostic nomogram are shown in Figure 4. These analyses thoroughly assessed the model’s predictive performance and diagnostic accuracy.

### 3.3 Comparison of performance among VOIs

**Figure 5:**
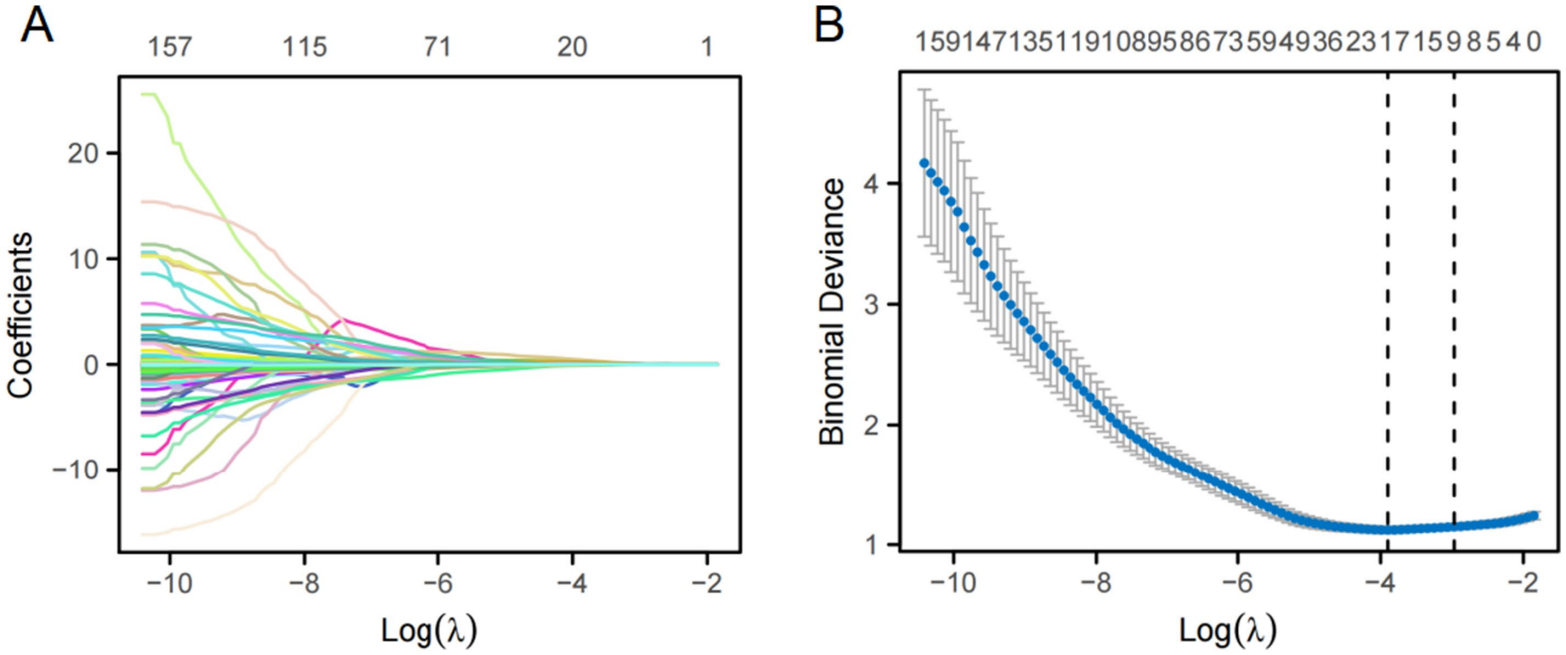
LASSO regression was used for feature selection. **(A)** LASSO coefficient selection: The optimal value of *λ* and the corresponding coefficients were identified using the coefficient plot. **(B)** LASSO variable trajectories: The feature importance plot was used to identify the most important features and the optimal value of λ.

In total, 1,688 radiomic features were extracted from each tumor and its surrounding tissues. The VOIs included intratumoral and peritumoral VOIs expanding 2, 4, 6, 8, and 10 mm from the tumor outline and their combinations. The ROC curve was used to evaluate the sensitivity and specificity of the nomogram (Figure 4). Among the radiomic-based predictive models for Ki-67 expression, the one based on combined-8 mm radiomic features demonstrated superior diagnostic performance (Tables 3, 4). This model was derived from features selected using LASSO and classified using a random forest algorithm (Figure 5). In the training set, its AUC, accuracy, sensitivity, and specificity for high Ki-67 expression were 0.883, 77.05, 90.7, and 72.04%, respectively. However, they were generally lower in the validation set (AUC = 0.820, accuracy = 80.73%, sensitivity = 63.16%, and specificity = 90.68%). After extensive analysis, combined-8 mm was determined to be the most predictive radiomic model, demonstrating its potential for clinical application in diagnosing meningiomas.

**Table 3:** Predictive model performance for Ki-67 expression in the intratumoral and peritumoral VOI.

Model	Training set				Internal validation set				External validation set			
	AUC (95% CI)	SEN (%)	SPE (%)	ACC (%)	AUC (95% CI)	SEN (%)	SPE (%)	ACC (%)	AUC (95% CI)	SEN (%)	SPE (%)	ACC (%)
Whole-Tumor	0.809 (0.761–0.857)	88.37	61.29	67.87	0.756 (0.700–0.812)	63.16	85.37	76.82	0.735 (0.673–0.797)	74.38	75.21	74.89
BTI-2 mm	0.768 (0.716–0.820)	97.67	49.46	58.61	0.727 (0.665–0.789)	68.42	75.61	73.17	0.637 (0.559–0.715)	77.14	59.65	65.28
BTI-4 mm	0.817 (0.771–0.863)	93.02	62.37	69.62	0.763 (0.711–0.815)	68.42	80.49	76.23	0.750 (0.694–0.806)	78.49	75.44	76.57
BTI-6 mm	0.785 (0.735–0.835)	67.44	81.87	76.68	0.749 (0.693–0.805)	57.89	87.80	75.45	0.654 (0.584–0.724)	71.43	66.67	68.40
BTI-8 mm	0.843 (0.801–0.885)	93.03	67.74	74.11	0.787 (0.739–0.835)	73.68	78.05	76.61	0.707 (0.649–0.765)	79.98	63.16	68.65
BTI-10 mm	0.756 (0.700–0.812)	67.44	74.19	71.91	0.675 (0.605–0.745)	94.74	43.90	52.89	0.673 (0.607–0.739)	65.71	75.44	71.42

AUC, area under the curve.

**Table 4:** Predictive model performance of the clinical, combined VOI, and clinical-radiomic models.

Model	Training set				Internal validation set				External validation set			
	AUC (95% CI)	SEN (%)	SPE (%)	ACC (%)	AUC (95% CI)	SEN (%)	SPE (%)	ACC (%)	AUC (95% CI)	SEN (%)	SPE (%)	ACC (%)
Clinical model	0.714 (0.648–0.780)	72.09	65.59	67.51	0.777 (0.715–0.839)	84.21	73.17	76.34	0.620 (0.535–0.705)	60.02	64.91	62.96
Combine-2 mm	0.809 (0.761–0.857)	74.42	76.34	75.72	0.779 (0.719–0.839)	73.68	80.49	78.20	0.682 (0.606–0.758)	85.71	52.63	61.69
Combine-4 mm	0.875 (0.839–0.911)	88.37	70.97	75.68	0.770 (0.706–0.834)	63.17	87.80	78.15	0.751 (0.689–0.813)	82.86	61.40	68.11
Combine-6 mm	0.806 (0.754–0.858)	79.07	75.27	76.43	0.792 (0.734–0.850)	68.42	85.37	79.16	0.701 (0.629–0.773)	74.29	66.67	69.38
Combine-8 mm	0.883 (0.851–0.915)	90.70	72.04	77.05	0.820 (0.770–0.870)	63.16	92.68	80.73	0.786 (0.734–0.838)	82.86	75.16	77.91
Combine-10 mm	0.830 (0.772–0.888)	88.37	68.82	74.00	0.743 (0.671–0.815)	73.68	75.61	74.99	0.766 (0.700–0.832)	88.54	63.16	70.89
Nomogram	0.904 (0.876–0.932)	83.72	84.95	84.56	0.877 (0.843–0.911)	84.21	82.93	83.33	0.837 (0.793–0.881)	88.57	71.31	77.02

AUC, area under the curve.

### 3.4 The diagnostic performance of the prediction models

In the ROC analysis, the radiomics-based model outperformed the clinical parameter-based model for predicting Ki-67 expression in both the training and validation sets. The clinical-radiomic model merged clinical predictors (peritumoral edema, heterogeneous enhancement) with the combined-8 mm radiomics features. Optimal predictive accuracy was achieved when the clinical and radiomic models were integrated, resulting in a clinical-radiomic model. In the training set, the integrated model had an AUC of 0.904, accuracy of 84.56%, sensitivity of 83.72%, and specificity of 84.95%. In the validation set, its performance was slightly lower but still robust, with an AUC of 0.877, accuracy of 83.33%, sensitivity of 84.21%, and specificity of 82.93%. Furthermore, in the external validation set, it maintained a high level of performance, with an AUC of 0.837, accuracy of 77.02%, sensitivity of 88.57%, and specificity of 71.31% (Table 4). These results indicate that the diagnostic model has better discrimination ability than the single radiomics-based and clinical parameter-based models. Additionally, the calibration curve for the nomogram, used for preoperative prediction of high Ki-67 expression in patients with meningioma, was highly concordant with actual outcomes, indicating that this prediction model has good reliability for preoperatively assessing Ki-67 expression.

### 3.5 Evaluation of the diagnostic nomogram through DCA

**Figure 6:**
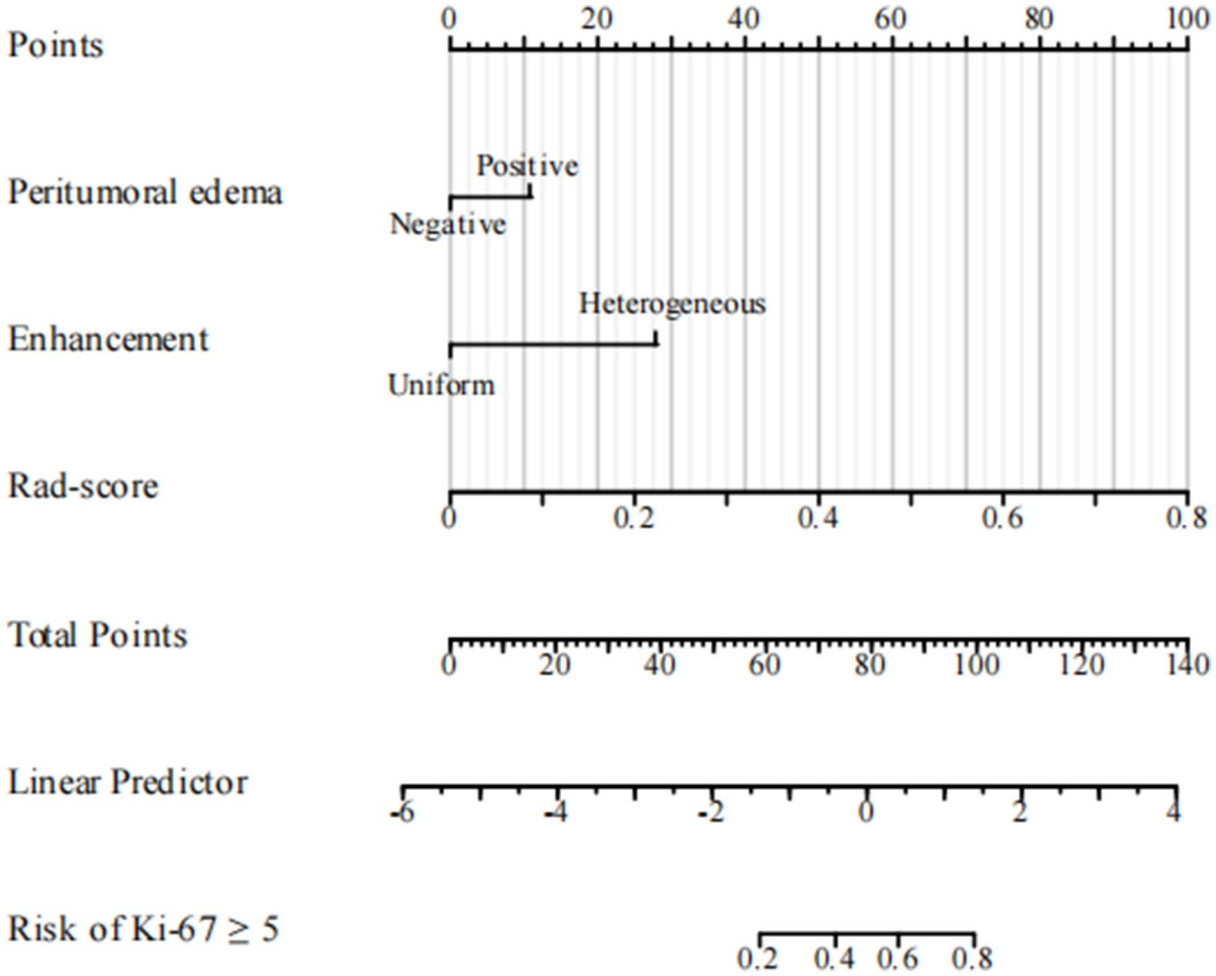
Diagnostic nomogram. The nomogram was constructed from peritumoral edema and contrast enhancement.

DCA was used to evaluate the diagnostic performance of the diagnostic nomogram and each single predictor model (Figure 6). The decision curve indicates the net benefit of patients when the intervention is performed under various threshold probabilities. The net benefit of the diagnostic nomogram model was the highest when the prediction model threshold probabilities were 0.21 ~ 0.66 (training set) and 0.58 ~ 1.09 (validation set), followed by the single radiomics-based model. Notably, the diagnostic nomogram model consistently provided a greater net benefit than the single predictor models, including the radiomics-based model. The example flowchart of prediction is shown in Supplementary Figure 1. The DCA and flowchart provided insights into the diagnostic performance of the prediction model.

## 4 Discussion

Our study revealed that meningioma with an elevated Ki-67 index was associated with an increased risk of peritumoral edema and heterogeneous enhancement, which are recognized indicators of brain invasion. We also demonstrated that VOI radiomics models based on CE-T1WI images, including intra- and peri-tumoral regions, can improve the accuracy in predicting Ki-67 expression levels in meningiomas. Then, we assessed the predictive performance of radiomics models with different VOI ranges for the peritumoral region. Among the examined radiomics feature ranges, the 8 mm was identified as the optimal peritumoral region. Then, we developed a combined clinical-radiomics model, integrating radiomics features from the intratumoral and 8 mm peritumoral regions. This integrated model demonstrated superior predictive efficacy. Ultimately, we developed an integrated clinico-radiomic model incorporating peritumoral edema, enhancement pattern, and tumor-peritumoral 8 mm radiomic features. This integrated model outperformed the individual radiomic and clinical models in terms of AUC, accuracy, and predictive efficacy for Ki-67 expression. The DCA and flowchart provide valuable insights into the diagnostic performance of the prediction model, enabling clinicians to make well-informed decisions regarding patient management strategies.

The Ki-67 expression is considered an indicator of the biological behavior of meningioma (18). Preoperative evaluation of Ki-67 expression can provide valuable supplementary information for clinical decision-making (19, 20). Systematic reviews have underscored the significance of accurately predicting Ki-67 status using radiological methods. For instance, a systematic review by Helal et al. (21) synthesized evidence on the accuracy and prognostic value of radiological predictions of Ki-67 in meningiomas, highlighting their potential to inform treatment strategies. Similarly, Broomand Lomer et al. (16) conducted a meta-analysis specifically on MRI-derived radiomics models, demonstrating their effectiveness in predicting Ki-67 index status. A higher Ki-67 index is generally associated with a more aggressive tumor phenotype and potentially poorer outcomes. Therefore, these findings suggest that patients presenting with these radiological features may require more intensive monitoring and treatment. However, since the Ki-67 assessment is susceptible to tumor heterogeneity, it is essential to evaluate the entire specimen, not only the core biopsy (22, 23).

MRI-based radiomics models have been reported to predict Ki-67 expression levels in meningiomas. Khanna et al. (12) predicted high Ki-67 expression in World Health Organization grade I meningioma using features extracted from multiple MRI sequences, with an AUC of 0.84 in their testing set. In Zhao’s study (11), the AUC for predicting the Ki-67 index in meningiomas was 0.837 in the internal validation set and 0.700 in the external validation set. These studies suggest that radiomics feature-based models can effectively predict both the grade of meningioma and the Ki-67 index. Both studies were based on intratumoral models. Recent advancements in MRI-based radiomics has demonstrated potential in preoperative prediction of meningioma Ki-67 expression. Li et al. (24) created machine learning models using multiparametric MRI to evaluate meningioma malignancy by WHO grading and prediction of Ki-67 index with AUCs of 0.92 and 0.87 in multicenter validation. Their study recognized the complementarity of clinical features and radiomics in stratification of tumor aggressiveness. Based on this, Duan et al. (25) developed a deep transfer learning radiomics nomogram that integrates multiparametric MRI features to make predictions of Ki-67 proliferation status with an AUC of 0.84 in external validation and highlights the technical advantages of combining domain-adaptive deep learning with traditional radiomics. Ouyang et al. (26) further advanced this field by constructing a contrast-enhanced MRI radiomics nomogram for Ki-67 prediction in two independent centers (AUC: 0.86), i.e., determining the clinical utility of texture features of arterial and venous phase images. While these studies together emphasize the diagnostic value of MRI radiomics in meningioma proliferation assessment, they are primarily founded on single-region tumor analysis. Previous research has indicated that peritumoral radiomics features offer greater insight into tumor heterogeneity. Our research builds upon previous studies by incorporating both intratumoral and peritumoral radiomics features into our predictive model. By integrating radiomics characteristics from the tumor core and its periphery, we aim to provide a comprehensive and encompassing assessment of the meningioma grade and Ki-67 index.

However, the peritumoral range for the VOI used in past radiological studies remains controversial. One study extracted imaging characteristics from various interfaces, including tumors and the brain (at distances of 1, 2, 3, 4, and 5 mm), establishing models that demonstrated superior generalization performance compared to current methods (27). Another study analyzed MRI radiomic features from intratumoral and peritumoral regions (at 5, 10, 15, and 20 mm distances) in a cohort of 92 patients with glioma. It found that models based on features from the 10 and 20 mm ranges were more effective at predicting Ki-67 levels and the expression of tumor protein p53 (TP53) (28). A further study involving 719 patients with meningioma revealed that including 4 mm peritumoral MRI radiomic features in the intratumoral model significantly improved diagnostic performance for meningioma invasion (29).

Evidently, a consistent criterion for the peritumoral boundary remains ambiguous. Therefore, we developed multiple VOI radiomics models with different peritumoral ranges to explore the distinct regions of highly aggressive meningioma. Considering that the actual tumor boundary extends beyond the image, we established five distinct peritumoral VOI ranges, extending the intratumoral VOI by 2, 4, 6, 8, and 10 mm. The VOI delineation methods for tumor and peritumoral regions with variable margins are illustrated in Supplementary Figure 2. Our results showed that the AUC for the 8 mm peritumoral region was 0.843 in the training set and 0.787 in the validation set. This region was identified as the most effective VOI, designating the 8 mm peritumoral area as the optimal selection. The radiomics model based on the 8 mm VOI encompassed the most predictive radiomics features.

Combining radiomics features from various categories resulted in the most robust predictive performance. While interpreting the complex relationship between pathophysiological processes and tumor structural features is challenging, tumors with greater structural heterogeneity are often more aggressive. Increased heterogeneity in meningiomas indicates a higher probability of infiltration into adjacent tissues. Greater heterogeneity is manifested in MRI as increased grayscale inhomogeneity and elevated image complexity. These features may indicate aggressive tumor behavior, such as breaching the tumor capsule and invading surrounding non-neoplastic tissues, which leads to increased intra- and peri-tumoral heterogeneity and reflects the underlying oncobiological and heterogeneity characteristics (30).

Integrating intratumoral and peritumoral radiological features offers superior advantages over relying exclusively on single-tumor radiomics, emphasizing the importance of combining intratumoral and peritumoral characteristics to enhance clinical insights (31). Further analysis revealed that the combined intratumoral and peritumoral 8 mm model achieved an AUC of 0.883 in the validation set, outperforming both the intratumoral model (AUC = 0.809) and the peritumoral 8 mm model (AUC = 0.843). This finding is consistent with Beig et al. (32), who demonstrated that lung adenocarcinomas and granulomas could be more effectively differentiated by combining radiomics features from within and around pulmonary nodules. With an AUC of 0.80, this integrated approach outperformed the single internal nodule model (AUC = 0.75), further emphasizing the importance of the peritumoral microenvironment in oncological research. These results highlight the need to consider the entire tumor environment for more accurate diagnostic and prognostic evaluations.

Regarding clinical factors, previous studies have demonstrated that characteristics such as tumor volume, tumor margin, and tumor-brain interface correlate with Ki-67 expression in meningioma (33), which is consistent with our findings. Pathological grade is a crucial prognostic factor in meningiomas (34), and there is a close correlation between grade and Ki-67 expression. High-grade pathology implies higher recurrence and worse prognosis. Existing studies have confirmed a significant correlation between Ki-67 expression and the invasion of pial/cortical and arachnoidal structures (35), and the Ki-67 proliferation index also serves as a predictor of recurrence timing (36). Our clinical model demonstrated only moderate predictive performance, suggesting that basic clinicopathological factors alone may not be sufficient.

Meticulous segmentation enables a thorough examination of the heterogeneity across multiple radiographic VOIs. Furthermore, there is an increased focus on the predictive value of Ki-67 expression in meningiomas, recognizing its established role as a proliferation marker closely associated with tumor aggressiveness and recurrence potential. By targeting this critical biomarker, the goal is not only identifying the optimal peritumoral VOI but also refining risk stratification strategies for patients. This dual focus highlights the contribution of our study in advancing both the scientific understanding and clinical management of meningioma.

Nonetheless, our study had several limitations. Firstly, its retrospective design and small sample size limited its power, highlighting the need for prospective studies with large sample sizes to verify our findings. Secondly, specific indicators of the tumor microenvironment, such as radiological features of immune cells, were not included in the developed model. Their inclusion could have provided a more comprehensive understanding of the relationship between radiological features and the peritumoral microenvironment. One of the major limitations of our research is the inhomogeneity of WHO grade data throughout the cohort. WHO grading data were not consistently available for all cases in our dataset. The WHO grading system represents a valuable pathological parameter with a well-documented association with Ki-67 expression, and its omission can restrict the generalizability and interpretability of our results. Future research including WHO grade data will be necessary for a more thorough assessment of the association between imaging characteristics and tumor biology.

## 5 Conclusion

In summary, this study developed innovative clinical-radiomic models to predict Ki-67 expression in meningiomas before surgery. By integrating radiomic features with clinical data, these models offer a novel non-invasive strategy for assessing tumor status, potentially enhancing the accuracy of preoperative evaluations and aiding in developing personalized treatment plans for patients with meningioma.

## Data Availability

The raw data supporting the conclusions of this article will be made available by the authors, without undue reservation.
