# Supplementary material for: MRI-based multiregional radiomics for preoperative prediction of Ki-67 expression in meningiomas: a two-center study
Source: Front Neurol. 2025 Jul 24;16:1554539. doi: 10.3389/fneur.2025.1554539 (PMC12328361; doi:10.3389/fneur.2025.1554539)

## MRI-based multiregional radiomics for preoperative prediction of Ki-67 expression in meningiomas: A two-center study

**Supplementary** **Tables**

**Table S1** The details of final selected features for each VOI

| *VOI | Number of features | | | |
| --- | --- | --- | --- | --- |
|  | First-order  features | ^#^Shape  features | Texture features | Total |
| Whole-Tumor | 1 | 2 | 6 | 9 |
| BTI-2mm | 3 | 0 | 10 | 13 |
| BTI-4mm | 1 | 3 | 7 | 11 |
| BTI-6mm | 3 | 1 | 11 | 15 |
| BTI-8mm | 2 | 2 | 9 | 13 |
| BTI-10mm | 1 | 0 | 10 | 11 |
| Combine-2mm | 2 | 1 | 9 | 12 |
| Combine-4mm | 4 | 1 | 10 | 15 |
| Combine-6mm | 3 | 2 | 7 | 12 |
| Combine-8mm | 5 | 2 | 11 | 18 |
| Combine-10mm | 3 | 1 | 12 | 16 |

*VOI-volume of interest

^#^The final selected shape features for each VOI had “shape_Flatness feature”

**Table S2** The details of final selected features for Combine-8mm_VOI

| Radiomics features | Coefficient |
| --- | --- |
| Whole-Tumor_lbp-3D-k_firstorder_Skewness | 0.017347784 |
| Whole-Tumor_original_glrlm_LongRunLowGrayLevelEmphasis | 0.041926023 |
| Whole-Tumor_logarithm_gldm_Median | 0.006598778 |
| Whole-Tumor_original_shape_MajorAxisLength | 0.118524505 |
| Whole-Tumor_wavelet-HLL_glszm_LargeAreaHighGrayLevelEmphasis | 0.027913502 |
| Whole-Tumor_wavelet-HLH_gldm_LargeDependenceLowGrayLevelEmphasis | 0.012810647 |
| Whole-Tumor_logarithm_firstorder_MeanAbsoluteDeviation | 0.023552069 |
| BTI-8mm_squareroot_firstorder_Median | 0.083055576 |
| BTI-8mm_original_glszm_LargeAreaLowGrayLevelEmphasis | 0.022919775 |
| BTI-8mm_logarithm_firstorder_Median | 0.011797019 |
| Whole-Tumor_lbp-3D-k_glszm_SizeZoneNonUniformity | 0.054250272 |
| BTI-8mm_squareroot_firstorder_Energy | -0.112383046 |
| Whole-Tumor_wavelet-HHL_glszm_HighGrayLevelZoneEmphasis | -0.034903733 |
| Whole-Tumor_wavelet-LLL_ngtd_Busyness | 0.019043281 |
| BTI-8mm_wavelet-LHL_gldm_DependenceVariance | -0.022038295 |
| BTI-8mm_wavelet-LHL_glszm_Kurtosis | 0.001462882 |
| BTI-8mm_original_shape_SurfaceVolumeRatio | 0.020301629 |

VOI-volume of interest

**Supplementary Figure 1** An example flowchart of prediction.


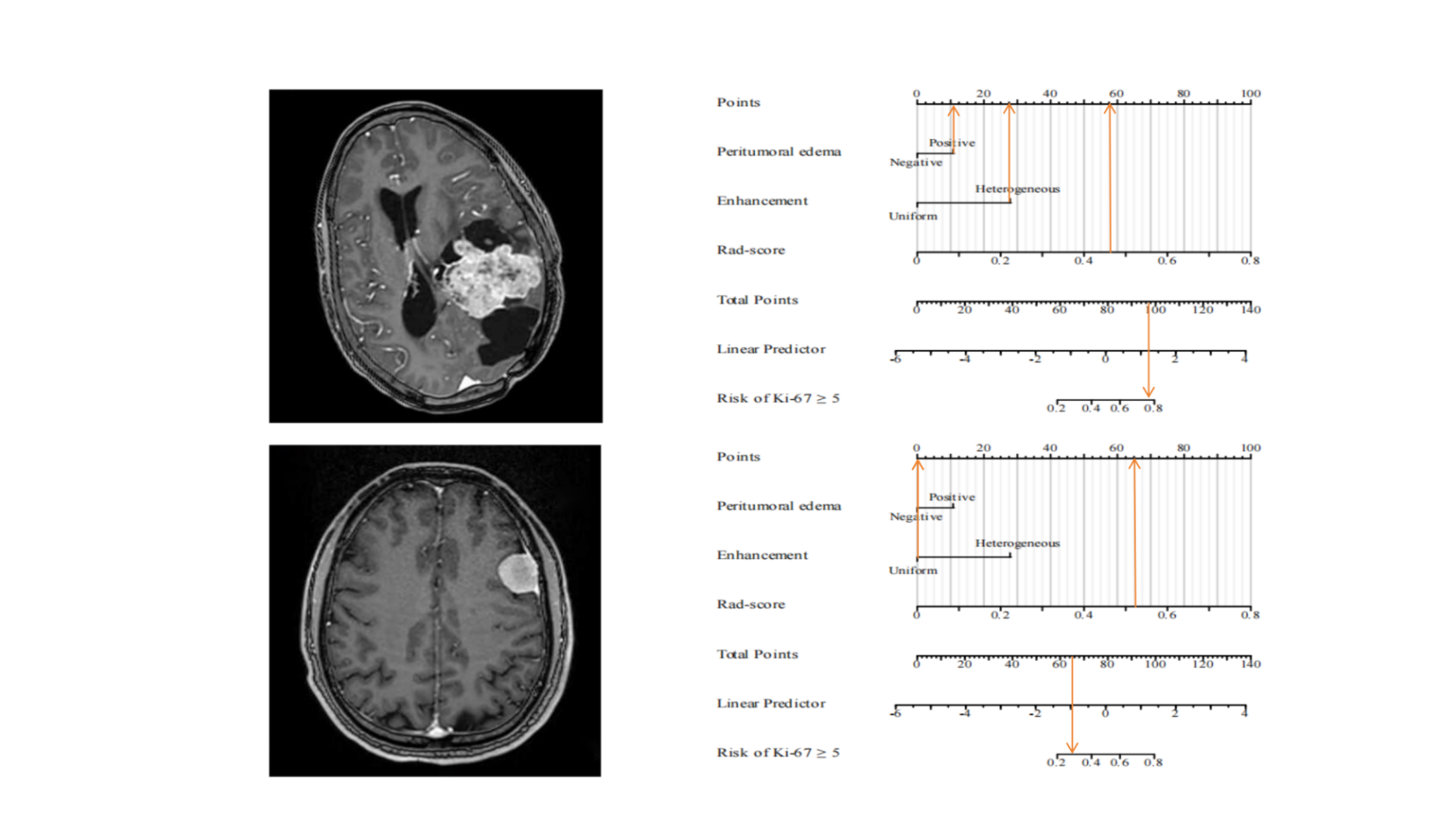


**Supplementary Figure 2** Illustration of VOI Delineation Methods for Tumor and Peritumoral Regions with Variable Margins.


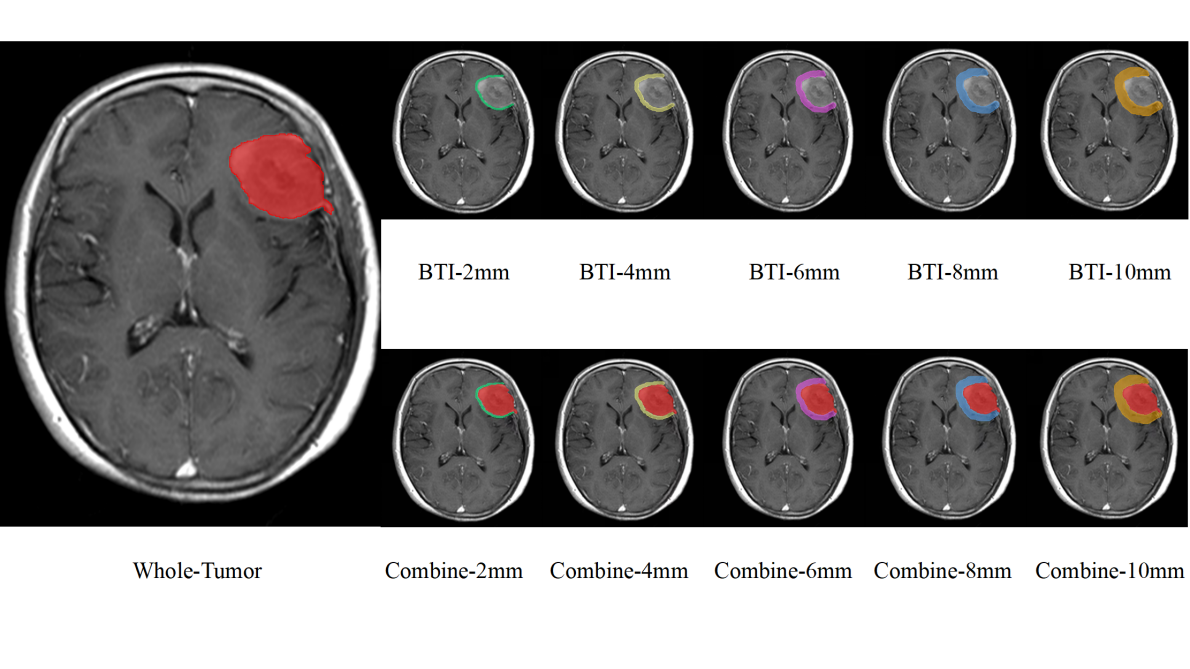

Supplement: Supplementary file 1 [file Supplementary_file_1.docx]
